# Supplementary material for: Nutritional Assessment in Inflammatory Bowel Disease (IBD)—Development of the Groningen IBD Nutritional Questionnaires (GINQ)
Source: Nutrients. 2019 Nov 12;11(11):2739. doi: 10.3390/nu11112739 (PMC6893781; doi:10.3390/nu11112739)
Supplement: Supplementary file 1 [file nutrients-11-02739-s001.zip › supplementary/Comparison of FFQs - Supplementary file 2.pdf]

Supplementary table 2. Comparison of FFQs

Main category	Food items	Comparison of questionnaires					
		DHQ III[1]	Harvard[2]	NNS[3]	Australia[4]	Iran[5]	GINQ[6]
Breakfast products	Breakfast drinks (readymade)[+]	x					x
Breakfast grains	Breakfast cereals	x	x	x	x		x
	Porridge grains	x	x	x	x		x
	Processed grains			x	x		x
Dairy	Milk[+]	x	x	x	x	x	x
	Milk alternatives[+]		x	x	x	x	x
	Chocolate milk	x					x
	Sweetened dairy drinks			x	x		x
	Health-improving dairy	x					x
	Yogurt[+]	x	x	x		x	x
	Quark[+]					x	x
	Dutch custard						x
	Ready-made porridge / pudding / dessert	x					x
	Porridge / pudding				x		x
	Sweetners added to yogurt / quark / etc						x
	Ice cream products	x	x	x	x		x
	Hard cheeses[+]	x	x	x	x	x	x
	Soft cheeses[+]	x	x	x	x	x	x
	Cream[+]	x	x	x	x	x	x
Bread and bread substitutes	Rusk / biscuit	x	x	x			x
	Knäckebröd						x
	Breakfast crackers/crispbread				x		x
	Croissants				x		x
	Hard rolls/ rustic rolls[+]	x	x	x	x		x
	Buns/rolls[+]	x	x	x	x		x
	Bread slices/sandwich bread[+]	x	x	x	x	x	x
Fats/oils	Butter spreads[+]	x	x		x	x	x
	Cooking fats/oils	x	x		x	x	x
Spreads	Nut pastas[+]	x	x	x	x		x
	Sweet spreads	x	x	x	x	x	x
	Savory (salad/humus) spreads[+]	x		x	x	x	x
Eggs	Fried eggs[+]	x	x	x			x
	Boiled eggs[+]	x	x	x			x
Fruits	Dried fruits[+]	x	x		x	x	x
	Fresh fruits[+]	x	x	x	x	x	x
	Processed fruits / Preserved fruits[+]	x	x		x		
Nuts, stone fruits, seeds	Nuts, stone fruits[+,°]	x	x	x	x	x	x
	Peanuts[+,°]	x	x			x	x
	Seeds[+,°]						x
Meat and meat substitutes	Raw cold cuts[+]						x
	Processed cold cuts[+]	x	x	x	x		x
	Meat[+]	x	x	x	x	x	x
	Gravy	x					x
	Meat substitutes	x			x		x
Fish	Fresh fish[+]	x	x	x	x	x	x
	Raw fish[+]	x			x		
	Processed fish[+]			x			
	Shellfish[+]	x		x	x		x
Vegetables	Vegetables[+]	x	x	x	x	x	x
	Raw vegetables[+]	x	x	x	x	x	x
Legumes	Legumes[+]	x	x	x	x	x	x
Grain products	Pasta[+]	x	x	x	x	x	x
	Rice / other grains[+]	x	x	x	x	x	x
	Potatoes[+]	x	x	x		x	x
	Toast, bread, wraps, pita, baguette, etc[+]	x	x		x	x	
Sauces	Warm sauces				x		x

	Dressing[+]	x	x	x	x		x
	Red sauces[+]	x	x		x	x	x
	Mayonnaise and other non-red sauces[+]	x	x		x	x	x
Additions to meals	Super foods[+]						x
	Seasoning[+]						x
	Spices & herbs[+]					x	x
	Spicy						x
	Olives / bell peppers[+]						
Fast food	Fast food[+]	x	x	x	x		x
Soup	Soup[+]	x	x				x
Savory snacks	Chips / pretzels	x	x	x	x		x
	Fried snacks				x		x
Sweet snacks	Chewing gum				x		x
	Dutch licorice						x
	Sweets/candy		x		x	x	x
	Bonbon	x					x
	Chocolate	x	x	x	x	x	x
	Candy bars	x	x				x
	Gingerbread[+] / cookie bars / nutritional biscuits	x	x		x		x
	Cake / large biscuits	x	x	x	x	x	x
	Small cookies / biscuits	x	x		x	x	x
	Pastry / pie	x	x	x	x		x
Beverages	Fruit juice	x	x	x	x	x	x
	Vegetable juice	x		x	x		x
	Tap water	x		x			x
	Bottled water / sparkled water	x	x				x
	Coffee[-]	x	x	x	x	x	x
	Tea	x	x	x	x	x	x
	Coffee milk	x	x	x			x
	Sweeteners[+]	x	x	x	x	x	x
	Soda[-]	x	x		x		x
	Lemonade	x	x	x	x	x	x
	Sport drinks	x	x				
	Energy drinks	x					x
	Protein shakes	x					
	Alcoholic beverages	x	x	x	x		x

1. Diet History Questionnaire III (DHQ III) - https://epi.grants.cancer.gov/dhq3/

2. FFQ of Harvard University - https://regepi.bwh.harvard.edu/health/FFQ/files/2007%20BOOKLET%20FFQ.pdf

3. FFQ of Australian Bureau of Statistics - National Nutrition Survey 1995- https://www.abs.gov.au/websitedbs/d3310114.nsf/4a256353001af3ed4b2562bb00121564/b5243fb56a17a812ca2572030023fa

4. FFQ of Monash University Australia - pdf

5. FFQ of Research Institute Shahid Beheshti University of Medical Sciences Iran - pdf

6. Groningen IBD Nutritional Questionnaires (GINQ-FFQ)

[+] *Added to meals (breakfast, lunch, dinner) (or beverages) or eaten as snack in between meals*

[°] *Salted or unsalted*

[-] *Caffeinated or decaffeinated*
